# Management of *Acinetobacter baumannii* infection in intensive care units

**DOI:** 10.7196/AJTCCM.2021.v27i1.143

**Published:** 2021-03-09

**Authors:** R Raine, K Dheda

**Affiliations:** 1 Division of Pulmonology, Department of Medicine and UCT Lung Institute, University of Cape Town, South Africa; 2 South African MRC/UCT Centre for the Study of Antimicrobial Resistance, University of Cape Town, South Africa; 3 Faculty of Infectious and Tropical Diseases, Department of Infection Biology, London School of Hygiene and Tropical Medicine, London, UK

## Editorial

Multidrug-resistant organisms (MDRO) are very common and troublesome accompaniments of treating patients in intensive care units. Invasive treatment modalities (endotracheal tubes, intravascular and urinary catheters, among others), broad-spectrum antibiotic use and comorbidities are major contributing factors to colonisation and infection. Treatment of MDRO is often very difficult.

Maasdorp *et al*.^[1]^ have shown that the combination of colistin and tigecycline is considerably more effective in treating *Acinetobacter baumannii* (*A. baumannii*) infection than colistin alone in a retrospective cross-sectional case series. The number of patients enrolled in the study was small (16 in total), but there was considerably better ICU survival in the colistin-tigecycline treatment group (77.8%) than the colistin only group (28.6%), with survival to hospital discharge being 55.6% and 28.6%, respectively.

*A. baumannii* is an organism with apparent variable pathogenicity, implying that treatment is not always necessary. The distinction between colonisation (with no need for antibiotic treatment) and invasive infection is very important. This study excluded patients with colonisation on the basis of absence of fever and other markers of active infection. Ntusi *et al*.^[2]^ reported on a cohort of patients admitted to the intensive care units at Groote Schuur Hospital and identified 38 patients out 251 (15.1%) with positive cultures for *A. baumannii* as having colonisation. Bloodstream infection was present in over 70% of patients who died in this cohort and was an independent predictor of mortality.^[2]^ Other risk factors for mortality included age, HIV-reactivity, low CD4 count, and multiple organ dysfunction occurring after onset of *A. baumannii* infection. Bloodstream infection was present in 44% of the Maasdorp cohort, but there was no apparent association with mortality, though this might reflect the small sample size. The challenge, and one that exists with many infections, is to identify patients with colonisation v. disease. That *A. baumannii* can be associated with severe pneumonia in a community-based setting,^[3]^ its association with poor prognostic markers that are clinically meaningful, and a clear response to treatment in some individuals suggest that ignoring this organism, when identified, is an unsatisfactory approach (and which has been adopted by some clinicians). The identification of biomarkers that portend disease rather than colonisation, in large prospective studies, is an urgent unmet need in the field.

Antibiotic treatment of infection is important, but is only one component of the complete management of MDRO infection. Measures to prevent the development and spread of MDRO are essential given the limited availability of effective antibiotics, particularly in an era when carbapenem-resistant enterobacteriaceae are becoming a greater problem than *A. baumannii*.

Measures to prevent the spread of MDRO were discussed in a previous editorial.^[4]^ Infection prevention and control measures that are essential include hand-hygiene, contact precautions, patient isolation or cohorting, and the use of bundles for ventilator management and invasive procedures.^[5,6]^ An additional issue is tracing the source of infection. Often this may be tracked to fomites or specific intrahospital locations such as casualty wards through which patients enter the hospital, surgical theatres, sluice room sinks etc. More studies are therefore required, including those using molecular fingerprinting and whole genome sequencing techniques^[7]^ to investigate the epidemiology and trace the source of *A. baumannii* within hospital settings.

In the meanwhile, strict compliance with infection prevention and control techniques for all intensive care units is important to attempt to alleviate the problem. Antimicrobial stewardship (AMS) is also essential. The main tenets of AMS are use of appropriate antibiotics in the appropriate dose for the appropriate duration, when antibiotics are needed.^[8]^ This study is a small step towards identifying the right antibiotics but requires a much larger investigation to confirm these findings. More studies in Africa, and also in HIV-infected persons together with extended drug-susceptibility profiling of *A. baumannii* isolates, are urgently required.
